# Supplementary material for: The Systems Biology Research Tool: evolvable open-source software
Source: BMC Syst Biol. 2008 Jun 29;2:55. doi: 10.1186/1752-0509-2-55 (PMC2446383; doi:10.1186/1752-0509-2-55)
Supplement: Additional file 1 — SBRT Archive. An archive of the current version of the Systems Biology Research Tool. [file 1752-0509-2-55-S1.zip › sbrt-1.4.0/doc/developers_guide/api/sbrt/ext/R/stat/class-use/RCorrelator.html]

Uses of Class sbrt.ext.R.stat.RCorrelator


|  |  |  |
| --- | --- | --- |
| **Overview** \| **Package** \| **Class** \| **Use** \| **Tree** \| **Deprecated** \| **Index** \| **Help** | |  |
| PREV   NEXT | **FRAMES**    **NO FRAMES**     **All Classes** |  |


---


## **Uses of Class sbrt.ext.R.stat.RCorrelator**

| Packages that use RCorrelator | |
| --- | --- |
| **sbrt.ext.R.stat** | Provides classes that allow communication with R for the purposes of statistics. |
| **sbrt.shell.mng.stat** | Provides classes and interfaces for creating statistical process managers. |

Uses of RCorrelator in sbrt.ext.R.stat

| Subclasses of RCorrelator in sbrt.ext.R.stat | |
| --- | --- |
| `class` | `RKendall<V>`             This class computes Kendall tau values using R. |
| `class` | `RPearson<V>`             This class computes Pearson r values using R. |
| `class` | `RSpearman<V>`             This class computes Spearman rho values using R. |

Uses of RCorrelator in sbrt.shell.mng.stat

| Methods in sbrt.shell.mng.stat that return RCorrelator | |
| --- | --- |
| `static RCorrelator<java.lang.String>` | `CorrelationManager.parseCorrelator(java.lang.String coeff)`             Returns the correlation computer corresponding to the provided string value. |

---


|  |  |  |
| --- | --- | --- |
| **Overview** \| **Package** \| **Class** \| **Use** \| **Tree** \| **Deprecated** \| **Index** \| **Help** | |  |
| PREV   NEXT | **FRAMES**    **NO FRAMES**     **All Classes** |  |


---
